# Suppression of PAX6 promotes cell proliferation and inhibits apoptosis in human retinoblastoma cells

**DOI:** 10.3892/ijmm.2014.1812

**Published:** 2014-06-17

**Authors:** BO MENG, YISONG WANG, BIN LI

**Affiliations:** 1 Beijing Institute of Ophthalmology, Beijing Tongren Hospital, Capital Medical University, Beijing 100005, P.R. China; 2 Department of Microbiology, School of Basic Medical Sciences, Capital Medical University, Beijing 100069, P.R. China

**Keywords:** PAX6, apoptosis, cell cycle, retinoblastoma

## Abstract

The aim of this study was to investigate the role of the transcription factor, PAX6, in the development of retinoblastoma. The expression of endogenous PAX6 was knocked down using PAX6-specific lentivirus in two human retinoblastoma cell lines, SO-Rb50 and Y79. Cell proliferation functional assays and apoptotic assays were performed on the cells in which PAX6 was knocked down. The results revealed that PAX6 knockdown efficiency was significant (P<0.01, n=3) in the SO-Rb50 and Y79 cells. The inhibition of PAX6 reduced tumor cell apoptosis (P<0.05, n=3), but induced cell cycle S phase arrest (SO-Rb50; P<0.05, n=3) and G2/M phase arrest (Y79; P<0.05, n=3). Western blot analysis indicated that the inhibition of PAX6 increased the levels of the anti-apoptotic proteins, Bcl-2, proliferating cell nuclear antigen (PCNA) and CDK1, but reduced the levels of the pro-apoptotic proteins, BAX and p21. In conclusion, our data demonstrate that the suppression of PAX6 increases proliferation and decreases apoptosis in human retinoblastoma cells by regulating several cell cycle and apoptosis biomarkers.

## Introduction

PAX6 is a member of the *PAX* gene family and encodes a conserved transcription factor with two DNA-binding domains, a paired domain and a paired-type homeodomain. PAX6 serves as a regulator in the coordination and pattern formation required for retinogenesis and the development of other ocular tissues (1,2). A number of previous studies have revealed the mechanisms involved in the transcriptional control of PAX6. For example, PAX6 has been found to bind to the proximal region of the tartrate acid phosphatase (TRAP) gene promoter and to suppress nuclear factor of activated T cells c1-induced TRAP gene expression (3). Recently, the upregulation of PAX6 has been observed in a number of ghrelin-expressing endocrine cells and plays an essential role in the adult maintenance of glucose homeostasis and function of the endocrine pancreas (4).

However, PAX6 has been found to be uniquely required for eye development. In the retina, PAX6 is involved in the regulation of the development of retinal progenitor cells into neurons and glial cells. As previously demonstrated, mice which were heterozygous carriers of a loss-of-function allele of PAX6 had defective eye development, while the homozygotes died after birth with defects in the eyes and brain (5–7). PAX6 has been found to initiate the multipotency of retinal progenitor cells. The inactivation of PAX6 restricts the multipotent potential of retinal progenitor cells, allowing them to generate only into amacrine interneurons (8). Furthermore, PAX6 has been shown to directly control the activation of retinogenic basic helix-loop-helix (bHLH) factors, influencing the differentiation of a subset of retinal progenitor cells. Emerging evidence has indicated that retinoblastoma tumors develop from embryological retinal photoreceptors (9,10). However the physiological role of PAX6 in retinal development and the oncogenesis in retinoblastoma remains largely unknown. The study by Xu *et al* demonstrated that retinoblastoma cells express markers of postmitotic cone precursors, and mouse double minute 2 (MDM2) and N-Myc are required for the proliferation and survival of these cells (11). They further demonstrated MDM2 expression is regulated by the cone-specific transcription factors, indicating the potential function of cone-specific signaling circuitry in the oncogenic effects of RB1 mutations.

Previous studies have indicated that the normal development of the mammalian eye is dependent on the level of PAX6 and insufficient expression levels of PAX6 lead to pan-ocular disorders, such as aniridia (12,13). We have previously demonstrated that the overexpression of PAX6 regulates the growth and apoptosis of human retinoblastoma cells (14,15). However the limitation of our previous studies exists in the phenotypes with increased copies number of PAX6, which may parallel with the phenotypes of a PAX6 haploinsufficiency. Therefore, in the present study, we suppressed the expression of Pax6 in human retinoblastoma cells and examined the effects on cell growth and apoptosis. The endogenous PAX6 knockdown was mediated by specific lentiviral PAX6-RNAi and validated by quantitative reverse transcription-polymerase chain reaction (RT-qPCR) and western blot analysis. The effects of the suppression of PAX6 on cell proliferation, cell cycle arrest and apoptosis were examined by fluorescence-activated cell sorting. The levels of apoptosis-related and cell cycle-related genes and proteins were detected by RT-qPCR and western blot analysis.

## Materials and methods

### Cell lines

Two human retinoblastoma cell lines, SO-Rb50 and Y79, were used in this study. The SO-Rb50 cell line was established in the Zhongshan Ophthalmic Center, Sun Yat-Sen University, Guangzhou, China, as previously described (15,16). The Y79 cell line was obtained from the American Type Culture Collection (ATCC; Manassas, VA, USA). The maintenance of these cell lines was carried out as previously described (17–19). In brief, the cells were cultured in RPMI-1640 medium (HyClone Co., Logan, UT, USA) supplemented with 10% fetal bovine serum, 100 U/l penicillin, and 100 U/l streptomycin at 37°C in a humidified atmosphere of 95% air/5% CO_2_. The culture medium was replaced every 2 days.

### Plasmids

A third generation of the self-inactivating lentiviral vector containing a cytomegalovirus (CMV) promoter-driven enhanced green fluorescence protein (eGFP) reporter was purchased from GeneChem Co., Ltd. (Shanghai, China). The lentiviral vector system was made from 3 types of plasmids, the pGCL-GFP vector (5′LTR, 3′LTR and woodchuck hepatitis virus post-transcriptional regulatory element), the pHelper 1.0 (gag, pol and rev element) vector and the pHelper 2.0 (VSV-G element) vectors. The pGCL-GFP vector encoding a sequence targeting the human *PAX6* gene (NCBI Reference Sequence ID: NM_000280.3) was assembled by GeneChem. PCR and DNA sequencing confirmed the accurate insertion of the small interfering RNA (siRNA) sequences. For the preparation of the recombinant lentiviral vectors, the pHelper 1.0 plasmid (15 μg), the pHelper 2.0 plasmid (10 μg) and the pGCL-GFP-siRNA or the pGCL-GFP plasmid (negative control, 20 μg) were co-transfected into subconfluent 293T cells in serum-free medium using Lipofectamine 2000 reagent (Invitrogen Co., Carlsbad, CA, USA). Untransfected cells were used as blank controls. After 8 h of incubation, the medium was changed to serum-containing medium. High titers of recombinant lentiviral vectors with PAX6-RNAi were harvested after the supernatant became concentrated 48 h later. Before the experiments, 7 human PAX6-specific double-stranded, siRNA sequences were synthesized and screened (Shanghai Genepharma Co., Ltd., Shanghai, China). We selected 2 sequences for co-transfection into the cell lines in our study. The target sites were CGTCCATCTTTGCTTGGGAAA and TACCAAGCGTGTCATCAATAA.

### Target site screening

Before the experiments, 7 human PAX6-specific double-stranded, siRNA sequences (KD1-KD7) were synthesized and screened. The details of the target sequences are presented in Table I. For transfection, the human retinoblastoma cells were seeded in 48-well plates at a concentration of 5×10^5^ cells/ml in a volume of 200 μl for each well. The cells were transfected either with PAX6-RNAi-GFP (PAX6 inhibition study group) or with GFP lentiviral vectors with scrambled siRNA (sequence: ‘TTCTCCGAACGTGTCACGT’) in serum-free medium for 20 h with a total multiplicity of infection (MOI) of 80. At 4 days after transfection, the reporter GFP gene expression was examined under a fluorescence microscopy. The transfected human retinoblastoma cell lines were screened using FACS for the following experiments.

### Cell proliferation

The standard colorimetric cell counting kit-8 (CCK-8; Dojindo Laboratories, Kumamoto, Japan) was used for the determination of the number of viable cells in the cell proliferation assays. The transfected retinoblastoma cells were seeded with a volume of 90 μl cell suspension (5,000 cells/well) into 96-well plates and 10 μl CCK-8 were added to each well followed by incubation for 4 h at 37°C. Optical densities were read using a microplate reader scanning at 450 nm. This procedure was repeated every 24 h during a 6-day period. Cell survival rates were measured at 3 time points of the cell growth curve through the log phase of growth for each cell line.

### RT-qPCR

Total RNA was extracted from the cells using TRIzol reagent [Tiangen Biotech (Beijing) Co., Ltd., Beijing, China; Cat. no. DP405]. The reverse transcription and the PCR amplification reactions were performed according to the M-MLV reverse transcriptase protocol (Tiangen Biotech) (Cat. no. KR104). qPCR (ABI PRISM 7500, version 1.41) was performed to detect the mRNA levels of PAX6 and cell cycle-related molecules in both retinoblastoma cell lines. The details of the primers for qPCR are presented in Table II and qPCR was carried out as previously described (20–22). The reaction system was 20 μl, including 4 μl RNase-free ddH_2_O, 4 μl cDNA template, 1.7 μl mixed primers, 10 μl SuperReal PreMix and 0.3 μl ROX Reference Dye l (Tiangen Biotech) (Cat. no. FP204). The PCR running conditions were as follows: 120 sec at 95°C for the initial denaturation followed by 45 cycles of 20 sec at 95°C for denaturation, 25 sec at temperature for annealing, and 30 sec at 95°C for extension. The threshold cycle (Ct value), which is the cycle number at which the amount of the amplified gene of interest reaches a fixed threshold, was subsequently determined. Relative quantification values of the target gene mRNA levels were normalized to endogenous human β-actin gene levels and calculated using the 2^−ΔΔCt^ method. Each experiment was performed 3 times.

### Apoptosis assay

The early apoptosis of the transfected cells was detected using the PE Annexin V Apoptosis Detection kit I (BD Pharmingen, San Diego, CA, USA) (Cat. no. 559763). The 2 retinoblastoma cell lines were washed twice with cold PBS and then re-suspended in 1× binding buffer at a concentration of 1×10^6^ cells/ml. A volume of 100 μl of the solution (1×10^5^ cells) was transferred to a 5 ml culture tube and 5 μl of PE Annexin V and 5 μl 7-AAD were added. The cells were gently dispersed and then incubated for 15 min at room temperature (25°C) in the dark. Finally, 400 μl of 1× binding buffer were added to each tube. The cells were analyzed using a BD FACSCalibur flow cytometer (BD Biosciences, San Diego, CA, USA) equipped with a 540 nm exciting laser. The results are shown as percentages of the count of the early apoptotic cells to the total cell count in the SO-Rb50 and Y79 retinoblastoma cell lines. Each experiment was performed 3 times.

### Cell cycle assay

A cell suspension corresponding to 1×10^6^ cells was collected and centrifuged. The supernatant was discarded, 1 ml of phosphate-buffered saline (PBS) at room temperature was added and the cell pellet was re-suspended. The full volume of re-suspended cells was transferred to 3 ml of absolute ethanol pre-cooled at −20°C by pipetting the cell suspension slowly into the ethanol while swirling at top speed. The cells were left in ethanol at −20°C for 15 min. The cells were centrifuged, the ethanol was discarded, 4 ml of PBS were added at room temperature and the cells were re-suspended to allow them to rehydrate for 15 min. Again, the cell suspension was centrifuged and the supernatant was discarded, 1 ml of DNA staining mixed solution was added and the cell suspension re-suspended. Following incubation for 30 min at room temperature, the cells were analyzed by FACS in the presence of the dye. The cells were then passed through a BD FACSCalibur flow cytometer equipped with a 488 nm argon laser to measure the DNA content. The data analysis was performed using Cell Quest (BD Biosciences) and ModFit LT software (Verity Software House Inc, Topsham, ME, USA) software. The results are presented as percentages of the total cell count in different phases of the cell cycle, namely the G0/G1 phase (diploid cells), S phase (diploid and tetraploid cells), and G2/M phase (tetraploid cells). Each experiment was performed 3 times.

### Western blot analysis

Approximately 1×10^7^ transfected human retinoblastoma cells of each of the 2 cell lines was collected into a 15 ml centrifuge tube and re-suspended in 600 μl cell lysis buffer [20 mM Tris (pH 7.5), 150 mM NaCl, 1 mM EDTA, 1% Triton X-100, 2.5 mM sodium pyrophosphate, 1 mM β-glycerophosphate, 1 mM Na_3_VO_4_, 1 μg/ml leupeptin, 1 mM phenylmethanesulfonyl fluoride (PMSF)]. The cells remained in that medium on ice for 30 min with re-dispersion every 5 min. The lysates were then centrifuged at 12,000 rpm for 5 min at 4°C. The protein concentrations were determined using a NanoPhotometer (Implen GmbH, München, Germany). The proteins were denatured in 5× loading buffer for 10 min and were separated by sodium dodecyl sulfate polyacrylamide gel electropheresis (SDS-PAGE) using 5% stacking and 12% separating gels. They were then electroplotted onto polyvinylidene difluoride (PVDF) membranes (0.2 μm, Immobilon-P; Millipore, Billerica, MA, USA) in the way of a semi dry process with 20 V for 30 min. After being blocked in a blocking solution (Beyotime Institute of Biotechnology, Beijing, China; Cat. no. P0023B) for 30–60 min, the membranes were incubated overnight at 4°C with primary antibodies, including mouse anti-human β-actin (diluted at 2,000; Beyotime Institute of Biotechnology; Cat. no. AA128), mouse anti-human PAX6 (diluted 500; Abcam Co., Hong Kong, China; Cat. no. Ab78545), rabbit anti-human cyclin-dependent protein kinase 2 (*CDK2*) (diluted at 1,000; Cat. no. 2546), mouse anti-human proliferating cell nuclear antigen (*PCNA*) (diluted at 1,000; Cat. no. 2586), mouse anti-human cyclin-dependent kinase 1 (*CDK1*) (diluted at 1,000; Cat. no. 9116), rabbit anti-human *Bcl-2* (diluted at 1,000; Cat. no. 2870) and rabbit anti-human *BAX* (diluted at 1,000; Cat. no. 5023) (all from Cell Signaling Technology, Inc., Beverly, MA, USA). After being rinsed in PBS solution with 0.5%v/v Tween-20 (PBST) thrice for 10 min, the PVDF membranes were incubated at 37°C for 1 h with the secondary goat anti-mouse IgG(H+L) antibody (diluted at 2,000) (Cat. no. A0216) or goat anti-rabbit IgG(H+L) antibody (diluted at 2,000) (both from Beyotime Institute of Biotechnology) (Cat. no. A0208) conjugated with horseradish peroxidase (HRP). Using PBST, the rinsed PVDF membranes were subjected to enhanced chemiluminescence (ECL) using an ECL detection kit (Beyotime Institute of Biotechnology) (Cat. no. P0018) and quantified using Quantity One software (Bio-Rad Laboratories Inc., Hercules, CA, USA). Each western blot analysis was performed 3 times. For further analysis, the means were calculated and the data were normalized.

### Statistical analysis

Statistical analysis was performed using a commercially available software package, SPSS (version 20.0 for Windows IBM; SPSS, Inc., Chicago, IL, USA). The distributions of the parameters were evaluated using the Levene test. The data are presented as the means ± standard deviation. Statistical analysis of the differences was carried out using an independent samples t-test and paired-samples t-test. A P-value of <0.05 was considered to indicate a statistically significant difference.

## Results

### Transfection efficiency of lentiviral vectors

**Figure 1:**
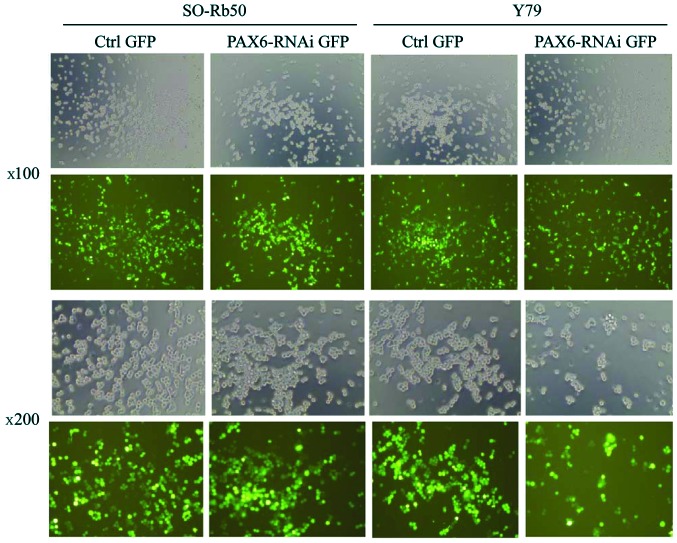
Transfection efficiency in the human retinoblastoma cell lines, SO-Rb50 and Y79, as detected by green fluorescence protein (GFP) using a fluorescence microscope at 4 days after transfection.

To investigate the function of PAX6 in both retinoblastoma cell lines, the gene was silenced by transfecting the retinoblastoma cells with lentiviral vectors carrying GFP-PAX6-RNAi sequences (PAX6 inhibition study group). The SO-Rb50 and Y79 cell lines were transfected with the lentiviral vectors at an MOI of 80. The successfully transfected cells expressed GFP and were examined under a fluorescence microscope at 4 days after transfection (Fig. 1). The efficiency of the infection was approximately 80% at an MOI of 80. The stably transfected cells were screened by FACS for the next step of the study.

### Target site screening

**Figure 2:**
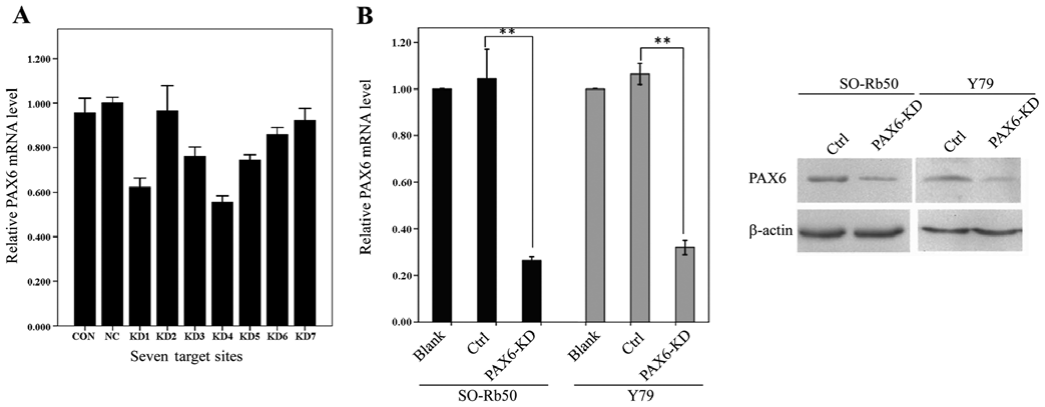
Screening and validating the putative knockdown sites of PAX6 by RT-qPCR. (A) Seven target sites (KD1-KD7) were analyzed by RT-qPCR in the SO-Rb50 cell line. KD4 showed the strongest inhibitory effect on PAX6 expression. (B) Inhibition of PAX6 by a combination of KD groups in the SO-Rb50 and Y79 cells. The mRNA level of PAX6 was significantly inhibited by the combination of KD4 + KD5 (^**^P<0.01, n=3). The protein level of PAX6 was determined by western blot analysis in the SO-Rb50 and Y79 cells.

Seven human PAX6-specific small interfering RNA sequences (KD1-KD7) were primarily screened using RT-qPCR in the SO-Rb50 cell line. The highest inhibition rate was 45% in KD4, indicating that the siRNA failed to have sufficient inhibitory effects (Fig. 2A). To obtain a higher inhibition rate, 2 combined target sites (KD1 + KD4, KD3 + KD4 and KD4 + KD5) were used to inhibit PAX6 in the 2 human retinoblastoma cell lines. The inhibition rate of the KD4 + KD5 group reached 70%, providing sufficeint inhibitory effects for the following experiments (Fig. 2B, left panel). The inhibitory effects on endogenous PAX6 expression were confirmed by western blot analysis. The protein levels of PAX6 in the SO-Rb50 and Y79 retinoblastoma cell lines were significantly lower in the knockdown groups than in the control groups (Fig. 2B).

### Suppression of PAX6 promotes cell proliferation

**Figure 3:**
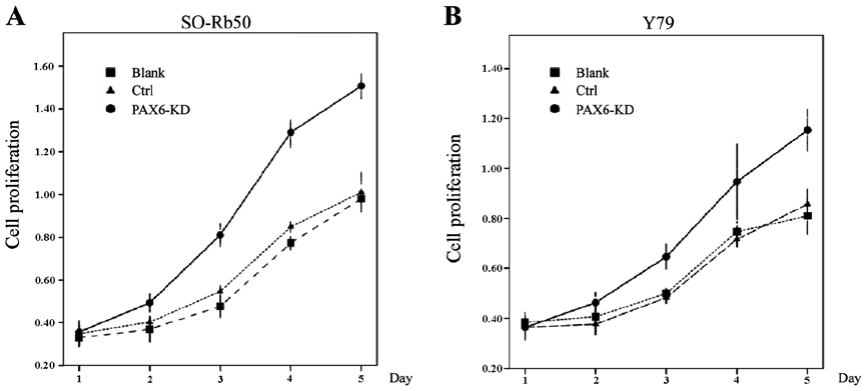
Suppression of PAX6 promotes cell growth. The OD value of (A) SO-Rb50 cells and (B) Y79 cells was determined 5 days after the inhibition of PAX6. Blank, without transfection; Ctrl, control, infected with control green fluorescence protein (GFP) lentivirus; PAX6-KD: infected with KD4 and KD5 lentivirus.

To examined the proliferation of the 2 retinoblastoma cell lines following the inhibition of PAX6, cell proliferation assay was performed using the standard colorimetric CCK-8. OD values were measured at 5 time points of the cell growth curve for each cell line. A significant increase in the cell survival rates was observed in the knockdown group in these 2 cell lines. The OD values of the 2 cell lines increased sharply at 1–5 days after cell plating in the knockdown group compared with the control groups (Fig. 3).

### Suppression of PAX6 inhibits apoptosis

**Figure 4:**
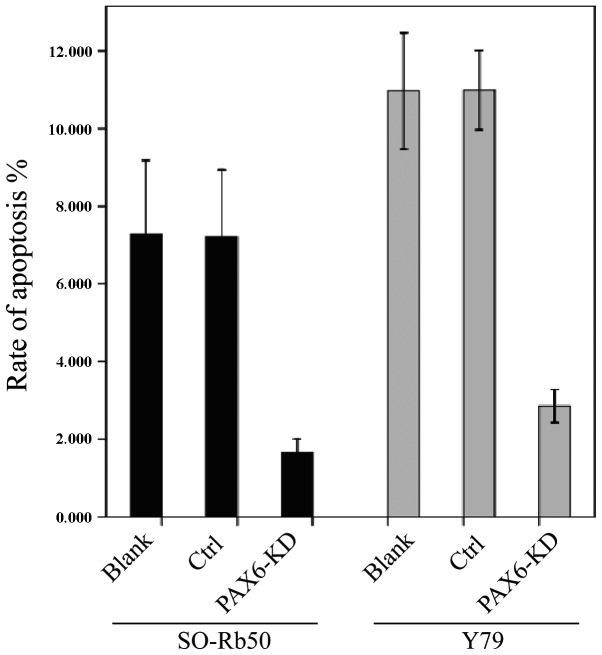
Suppression of PAX6 inhibits apoptosis. Early apoptosis assay was performed by FACS analysis in (A) SO-Rb50 cells and (B) Y79 cells. The percentage apoptotic cell counts in relation to the total cell counts were plotted in the form of stacked bar diagrams. Blank, without infection; Ctrl, control, infected with control green fluorescence protein (GFP) lentivirus; PAX6-KD, infected with KD4 and KD5 lentivirus.

The effects of endogenous PAX6 inhibition on cell apoptosis were examined using the PE Annexin V Apoptosis Detection kit I. Flow cytometric analysis revealed a reduced early apoptotic rate in the PAX6-knockdown groups compared to the control groups. The percentage of apoptotic cells was significantly lower in the PAX6-knockdown group than the corresponding negative GFP-control groups (t=4.036, P>0.05, n=3) and the negative control group without transfection (t=7.948, P<0.05, n=3) (Fig. 4).

### Suppression of PAX6 regulates cell cycle distribution

**Figure 5:**
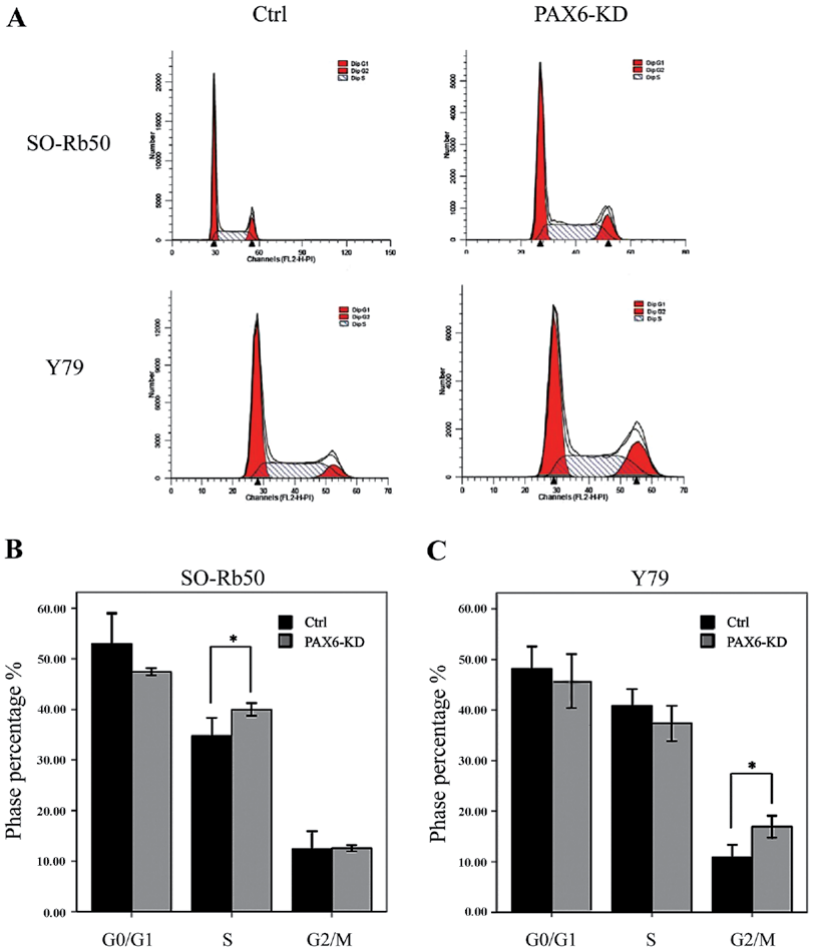
Suppression of PAX6 regulates cell cycle arrest. (A) Cell cycle analysis was performed by FACS in the SO-Rb50 cells and Y79 cells. Relative phase group was calculated by FACS in (B) SO-Rb50 cells and (C) Y79 cells (^*^P<0.05, n=3). Ctrl, contorl, infected with control green fluorescence protein (GFP) lentivirus; PAX6-KD, infected with KD4 and KD5 lentivirus.

We then determined the effects of the inhibition of endogenous PAX6 on the cell cycle by FACS. For the SO-Rb50 cell line, the percentage cell count in the S phase was significantly higher in the PAX6-knockdown group than in the negative GFP-control group (40.00±1.10 vs. 34.69 ± 3.17%; t=−4.44; P<0.05, n=3). For the the Y79 cell line, the percentage cell count in the G2/M phase was significantly higher in the PAX6-knockdown group than in the negative GFP-control group (16.92±1.89 vs. 10.78±2.23%; t=−8.31, P<0.05, n=3) (Fig. 5).

### Suppression of PAX6 affects cell cycle-related gene expression

**Figure 6:**
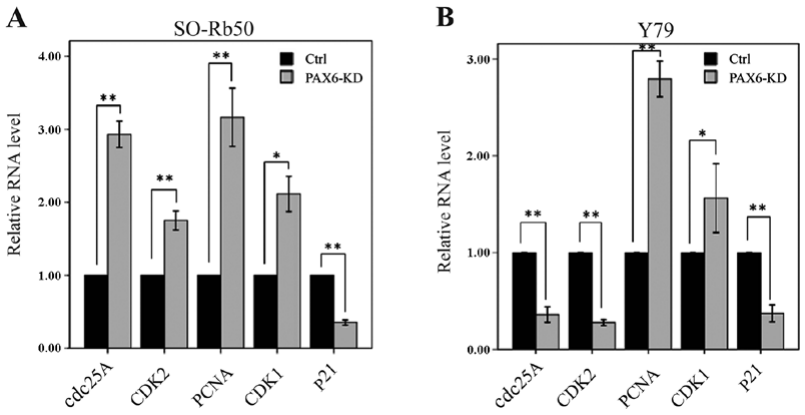
Suppression of PAX6 regulates cell cycle-related genes. The mRNA levels of cell cycle-related genes [cdc25A, cyclin-dependent protein kinase 2 (CDK2), proliferating cell nuclear antigen (PCNA), cyclin-dependent protein kinase 1 (CDK1) and p21] were measured by RT-qPCR in (A) the SO-Rb50 cells and (B) the Y79 cells (^*^P<0.05, ^**^P<0.01, n=3 ). Ctrl, control, infected with control green fluorescence protein (GFP) lentivirus; PAX6-KD, infected with KD4 and KD5 lentivirus.

To determine how the suppression of PAX6 affects cell cycle distribution, we measured the mRNA levels of cell cycle-related genes by RT-qPCR. In the SO-Rb50 retinoblastoma cell line, the mRNA levels of *cdc25A, CDK2, PCNA* and *CDK1* were significantly higher in the PAX6 inhibition study group than in negative GFP-control group (*cdc25A*, 2.93±0.16 vs. 1.00±0.00; t=−21.47; P<0.01, n=3; *CDK2*, 1.75±0.11 vs. 1.00±0.00; t=−11.56; P<0.01, n=3; *PCNA*, 3.16±0.35 vs. 1.00±0.00; t=−10.86; P<0.01, n=3; *CDK1*, 2.11±0.21 vs. 1.00±0.00; t=−9.22; P<0.05, n=3) (Fig. 6A). However, the mRNA level of *p21* was significantly lower in the PAX6 inhibition study group than in negative GFP-control group (0.35±0.03 vs. 1.00±0.00; t=37.25; P<0.01, n=3) (Fig. 6A). In the Y79 retinoblastoma cell line, the mRNA levels of *cdc25A, CDK2* and *p21* were significantly lower in the PAX6 inhibition study group than in negative GFP-control group (*cdc25A*, 0.36±0.07 vs. 1.00±0.00; t=15.91; P<0.01, n=3; *CDK2*, 0.28±0.03 vs. 1.00±0.00; t=46.77; P<0.01, n=3; *p21,* 0.37±0.08 vs. 1.00±0.00; t=14.37; P<0.01, n=3) (Fig. 6B). The mRNA levels of *PCNA* and *CDK1* were significantly higher in the PAX6 inhibition study group than in negative GFP-control group (*PCNA*, 2.79±0.16 vs. 1.00±0.00; t=−19.47; P<0.01, n=3; *CDK1*, 1.56±0.31 vs. 1.00±0.00; t=−3.15; P<0.05, n=3) (Fig. 6B).

### Suppression of PAX6 affects proteins related to apoptosis and the cell cycle

**Figure 7:**
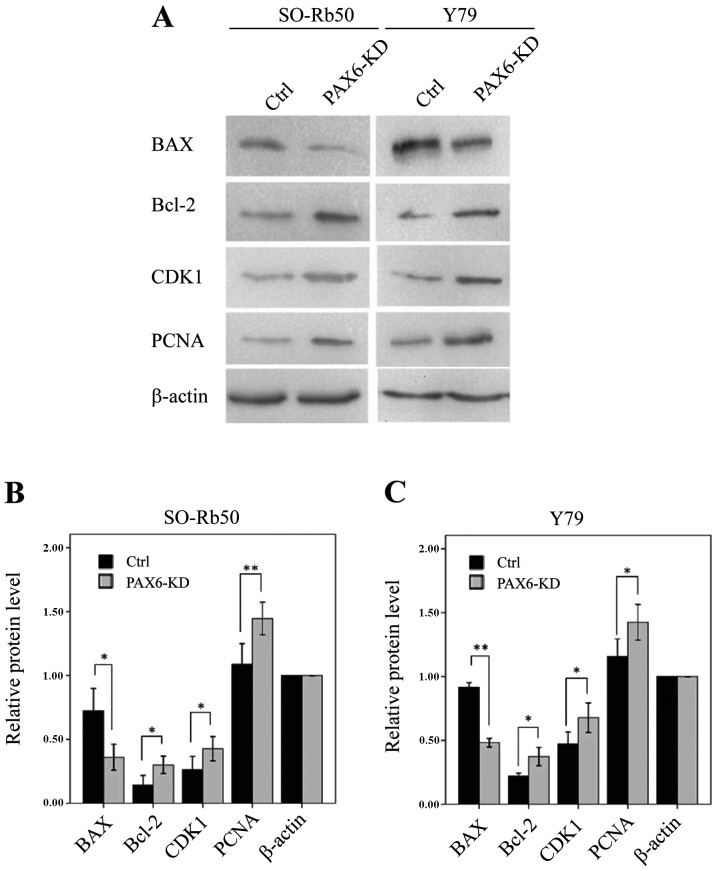
Suppression of PAX6 regulates cell cycle-related genes and apoptotic genes. (A) The protein levels of cell cycle-related genes [cyclin-dependent protein kinase 1 (CDK1) and proliferating cell nuclear antigen (PCNA)] and apoptotic genes (BAX and Bcl-2) were measured by western blot analysis in the SO-Rb50 and Y79 cells. Relative protein levels were quantified using Quantity One software in (B) the SO-Rb50 cells and (C) the Y79 cells (^*^P<0.05, ^**^P<0.01, n=3). Ctrl, control infected with control green fluorescence protein (GFP) lentivirus; PAX6-KD, infected with KD4 and KD5 lentivirus.

To determine the molecular mechanisms involved in the inhibition of PAX6 expression, as well as the signaling pathways associated with the cell cycle and apoptosis, we measured the expression of apoptosis-related *Bcl-2* and *BAX* and the cell cycle regulatory proteins, *CDK1* and *PCNA,* by western blot analysis. In both retinoblastoma cell lines, the levels of *Bcl-2, CDK1* and *PCNA* were significantly upregulated in the PAX6 inhibition study groups than in negative GFP-control groups (*Bcl-2,* t=−5.90, P<0.05 and t=−4.86, P<0.05, resp. n=3; *CDK1,* t=−5.27, P<0.05; and t=−7.60, P<0.05, resp. n=3; *PCNA*, t=−11.49, P<0.01; and t=−4.32, P=0.05, resp. n=3) (Fig. 7). However, the level of *BAX* was downregulated in both retinoblastoma cell lines (t=4.51, P<0.05; and t=67.96, P<0.01, resp. n=3) (Fig. 7).

## Discussion

Using a lentiviral vector-mediated transfection of human retinoblastoma cells, our study demonstrates that the inhibition of endogenous PAX6 expression results in decreased retinoblastoma cell apoptosis *in vitro*. First, we transfected 2 retinoblastoma cell lines with a lentiviral pGCL-GFP vector encoding a PAX6 siRNA sequence. The transfection was confirmed by measuring the GFP expression using a fluorescence microscope at 4 days after transfection (Fig. 1). We then confirmed the silencing effect on PAX6 by RT-qPCR. The results revealed that the mRNA levels of endogenous PAX6 were significantly lower in the PAX6-knockdown groups than in the control groups. Furthermore, we observed a phenotype with a reduced early apoptotic rate in the PAX6-knockdown groups by flow cytometric analysis. We also found that the percentage of cells in the S phase or the G2/M phase of the cell cycle was significantly higher in the PAX6-knockdown groups than in the control groups (Fig. 6). These changes in the cell cycle were paralleled with the changes in the mRNA levels of the cell cycle-associated genes, such as *cdc25A*, *CDK2*, *PCNA*, *CDK1* and *p21*. The upregulation of anti-apoptotic proteins and the downregulation of pro-apoptotic proteins was also confirmed by western blot analysis.

Apoptosis is an important biological phenomenon and plays a key role in organic evolution, the maintenance of the internal environment and the development of tumors (23). Proto-oncogenes and tumor suppressor genes associated with proliferation are found to participate in the regulation of apoptosis. These conserved genes contain the *Bcl-2* gene family, the caspase gene family and *p53*. The *Bcl-2* gene family is divided into two groups; one group includes inhibiting factors of apoptosis, such as *Bcl-2, Bcl-xL, Bcl-w* and *mcl-1*, while the other group includes apoptosis-promoting factors, such as *BAX, Bcl-xs, Bak* and *Bad*. The proportion of Bcl-2 and BAX determines the fate of the cells (24). In this study, we found that inhibition of PAX6 in human retinoblastoma cell lines led to a low apoptotic rate which was supported by the changes in the levels of apoptosis-related proteins (*Bcl-2 and BAX*). Consistently, Kashiwagi *et al* reported that cotylenin A induced apoptosis and inhibited cell proliferation by upregulating the mRNA expression of p21 and PAX6 in the retinoblastoma cell lines, Y79 and WERI-Rb1 (25). Ouyang *et al* reported that PAX6 suppressed the proliferation of corneal epithelial cells and led to apoptosis (26). Zhang *et al* reported that the inhibition of PAX6 blocked neuroectoderm specification from human embryonic stem cells (27). In the same manner, the overexpression of PAX6(a) has been shown to contribute to the differentiation of human embryonic stem cells. Davis *et al* found that the upregulation of PAX6 inhibited cell proliferation (28). The lack PAX6 in knockout models has been shown to increase cell proliferation in the cerebral cortex by shortening the cell cycle of progenitor cells at the onset of corticogenesis (29–31). Berger *et al* demonstrated that the activation of PAX6 inhibited proliferation and induced the apoptosis of cortical progenitors in the developing cortex (32).

However, the function of PAX6 in proliferation and apoptosis remains controversial. Certain studies have shown that a high expression of PAX6 is found in proliferating lens epithelial cells and that PAX6 expression is essential for maintaining the multipotency of retinal precursor cells (8,33–37). For instance, Maulbecker and Gruss found that PAX6 induced tumor formation in mice (9). Yamaoka *et al* demonstrated that the overexpression of PAX6 increased the proliferation of ductal epithelial cells in the mouse pancreas and led to the development of cystic pancreatic adenomas (38). In our previous study, we reported that the lentiviral vector-mediated overexpression of PAX6 in human retinoblastoma cells was associated with an increased cell proliferation parallel to a reduced caspase-3-dependent apoptotic rate, and with a change in the p53-regulated cell cycle (14). All these controversial data suggest that either an abnormally low level or high level of PAX6 is associated with a reduced rate of apoptosis and an increased rate of proliferation of (retinoblastoma) tumor cells. Furthermore, another study suggested that the quantitative and spatiotemporal expression of PAX6 influences its effect and functions (39). Accordingly, Zhang *et al* demonstrated that inducible PAX6(+5a) expression showed a biphasic and dose-dependent regulation of δ-catenin (a neural specific member of the armadillo/β-catenin superfamily) expression and cell fates. A moderate upregulation of Pax6(+5a) promoted δ-catenin expression and induced neurite-like cellular protrusions, but increasing the expression of PAX6(+5a) reversed these processes (40).

Apart from the decreased tumor cell apoptosis after the inhibition of endogenous PAX6 in the human retinoblastoma cells, we found that the inhibition of endogenous PAX6 led to an induction into the cell cycle S phase of the SO-Rb50 cell line and an induction into the cell cycle G2/M phase of the Y79 retinoblastoma cell line. Previous studies have suggested that PAX6 regulates cell proliferation and apoptosis by controlling the cell cycle in a cell type-specific manner. Duparc *et al* showed that PAX6 null retinal spheres over-proliferated and displayed reduced expression levels of several negative regulators of the cell cycle, such as p16, p27 and p21 (41). They also found that the gain of function of PAX6 suppressed cellular proliferation and secondary sphere formation. Accodingly, our present results indicated that the level of p21 was significantly lower in the PAX6-knockdown groups than in the control groups (Fig. 7). Similarly, other studies have shown that the suppression of PAX6 is associated with a lengthening of the cell cycle S phase (29,30,42). Shaham *et al* reported that PAX6 negatively regulates SOX2 [(sex determining region Y)-box 2] in the embryonic lens and suggested that PAX6 is a crucial factor in cell cycle exit (43). Ouyang *et al* reported that PAX6 overexpression retards the cell cycle in corneal epithelial cells (26). Hsieh *et al* demonstrated that maintaining a relatively low level of PAX6 is necessary for the S phase re-entry (5). All these data are in accordance with those of our study and support the hypothesis that PAX6 affects the proliferation and apoptosis of cells by regulating the cell cycle.

To determine the possible mechanisms of how PAX6 regulates cell cycle progression, we examined the mRNA and protein levels of several cell cycle regulatory genes, such as *cdc25A, CDK2, PCNA CDK1* and *p21*. In the PAX6-knockdown cells, CDK2 expression was increased in the SO-Rb50 retinoblastoma cells, suggesting an induction into the S phase. This finding was supported by an increased level of cdc25A, an upstream factor which promotes the expression of CDK2 and shortens the cell cycle. The mRNA level of PCNA was elevated in the PAX6-knockdown groups. PCNA is a DNA polymerase accessory factor which is involved in DNA replication and repair during the S phase of the cell cycle. In a parallel manner, the mRNA level of p21 was decreased in the study groups. p21 may block the ability of PCNA to bind with Gadd45 (44,45). At the G2/M checkpoint, CDK1 controlling the entry into the M phase is pivotal in regulating this transition. As an inhibitor of CDK1, p21 may be turned off by the activation of the CDK1/cyclin *B* complex (46–48). In our study, the level of CDK1 was upregulated and correspondingly, the level of p21 was downregulated in the study groups. These findings may explain the induction of tumor cell G2/M phase in the Y79 cells. Our results suggested that PAX6 was associated with the cell cycle through cdc25A/CDK2-dependent pathways and p53/p21/CDK1-dependent pathway. This was supported by parallel findings on the concentration of S phase- and G2/M phase-related proteins (PCNA and CDK) and apoptosis-related proteins (Bcl-2 and BAX).

In conclusion, our data demonstrate that the lentiviral-vector-mediated inhibition of endogenous PAX6 expression in human retinoblastoma cells is associated with an increased proliferation and a decreased apoptosis of human retinoblastoma cells *in vitro*, paralleled by corresponding changes in cell cycle-related and apoptosis-related molecules.

## Figures and Tables

**Table I:** Sequences of 7 target sites in PAX6.

Name	Sequences of target sites	Starting site	GC%
KD1	CGTCCATCTTTGCTTGGGAAA	817	47.62
KD2	CATGGCAAATAACCTGCCTAT	1541	42.86
KD3	GCAAGAATACAGGTATGGTTT	1287	38.10
KD4	taCCAAGCGTGTCATCAATAA	883	38.10
KD5	aaGATTCAGATGAGGCTCAAA	1117	38.10
KD6	gaGAGTAGCGACTCCAGAAGT	761	52.38
KD7	caCACCTAGTCATATTCCTAT	1373	38.10

**Table II:** Primers used for qPCR.

Gene	Gene ID	DNA sequences (5′→3′)	Tm (°C)
β-actin	NM_001101.3	TGGCACCCAGCACAATGAA	
		CTAAGTCATAGTCCGCCTAGAAGCA	58
PAX6	NM_000280	ATGGGCGGAGTTATGATACCTAC	
		GGAACTTGAACTGGAACTGACA	58
cdc25	NM_001789.2	CGTGGCTGCCTGCACTCTCA	
		GGCTGTCACAGGTGACTGGGG	60
CDK2	NM_001798.3	CCAGTACTGCCATCCGAGAG	
		CGGCGAGTCACCATCTCAGC	60
PCNA	NM_002592.2	CTGAGGGCTTCGACACTAC	
		TCACTCCGTCTTTTGCACAG	55
CDK1	NM_001786.4	AAGCCGGGATCTACCATACC	
		CCTGGAATCCTGCATAAGCAC	60
p21	NM_000389.4	GGACAGCAGAGGAAGAC	
		GGCGTTTGGAGTGGTAGAA	55

Tm, temperature; CDK2, cyclin-dependent protein kinase 2; PCNA, proliferating cell nuclear antigen; CDK1, cyclin-dependent protein kinase 1.
