# [Corrigendum] Suppression of PAX6 promotes cell proliferation and inhibits apoptosis in human retinoblastoma cells

**DOI:** 10.3892/ijmm.2025.5688

**Published:** 2025-11-10

**Authors:** Bo Meng, Yisong Wang, Bin Li

Int J Mol Med 34: 399-408, 2014; DOI: 10.3892/ijmm.2014.1812

Following the publication of this paper, it was drawn to the Editor's attention by an interested reader that, for the western blot experiments shown in Fig. 7A on p. 405, the Bcl-2 and PCNA blots for the SO-Rb50 cell line appeared to be identical, albeit it with possibly slightly different exposure time of the gel and different vertical dimensions. Similarly, the BAX and PCNA blots for the Y79 cell line also appeared to be identical, although the blots were rotated by 180° relative to each other, again with possibly slightly different exposure time of the gel and different vertical dimensions. In addition, for the experiments showing transfection efficiency in Fig. 1 on p. 402, the 'SO-Rb50/x100/PAX6-RNAi GFP' and 'Y79/x200/Ctrl GFP' data panels contained overlapping data, and the 'SO-Rb50/x200/PAX6-RNAi GFP' and 'Y79/x100/Ctrl GFP' data panels similarly contained overlapping data, suggesting that these pairings of panels had been placed in this figure the wrong way around.

Upon contacting the authors about these issues, they realized that Figs. 1 and 7 in this paper had inadvertently been assembled incorrectly. The revised versions of Fig. 1, now featuring the correct data for the PCNA blots for both the SO-Rb50 and the Y79 cell lines, and Fig. 7, now showing the correctly positioned data panels for the 'SO-Rb50/x100/PAX6-RNAi GFP' and 'Y79/x200/Ctrl GFP' experiments, are presented on the next page. The authors wish to emphasize that the errors made in assembling the data in these Figures did not affect the overall conclusions reported in the paper. The authors are grateful to the Editor of *International Journal of Molecular Medicine* for granting them this opportunity to publish a Corrigendum, and apologize to both the Editor and the readership for any inconvenience caused.

## Figures and Tables

**Figure 1:**
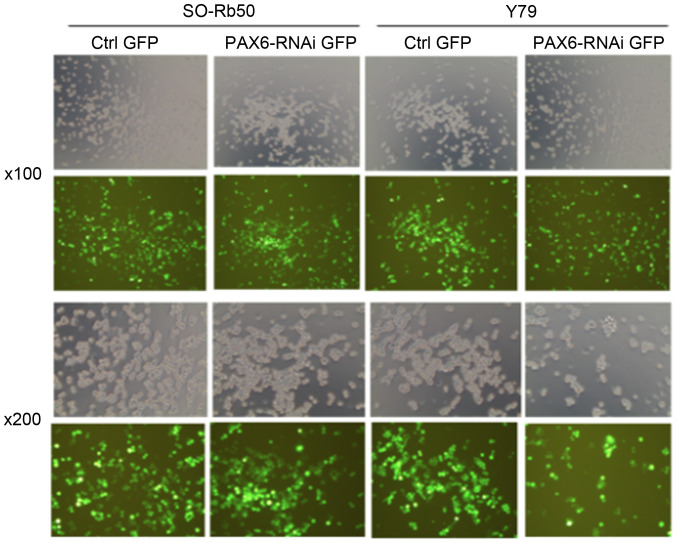
Transfection efficiency in the human retinoblastoma cell lines, SO-Rb50 and Y79, as detected by green fluorescence protein (GFP) using a fluorescence microscope at 4 days after transfection.

**Figure 7:**
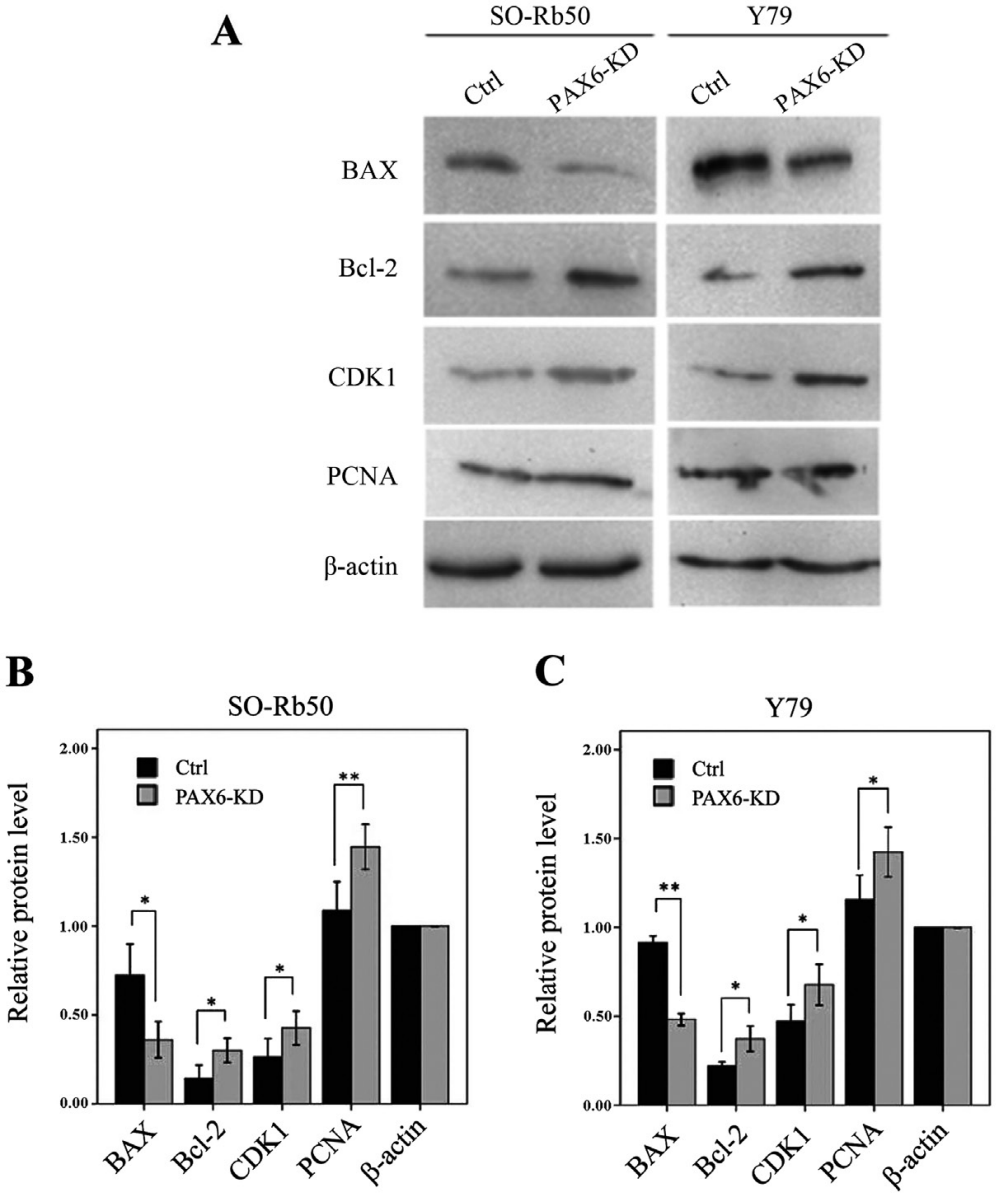
Suppression of PAX6 regulates cell cycle-related genes and apoptotic genes. (A) The protein levels of cell cycle-related genes [cyclin-dependent protein kinase 1 (CDK1) and proliferating cell nuclear antigen (PCNA)] and apoptotic genes (BAX and Bcl-2) were measured by western blot analysis in the SO-Rb50 and Y79 cells. Relative protein levels were quantified using Quantity One software in (B) the SO-Rb50 cells and (C) the Y79 cells (^*^P<0.05, ^**^P<0.01, n=3). Ctrl, control infected with control green fluorescence protein (GFP) lentivirus; PAX6-KD, infected with KD4 and KD5 lentivirus.

